# Comparative Genomics and In Vitro Experiments Provide Insight into the Adaptation and Probiotic Properties of *Shouchella clausii*

**DOI:** 10.3390/microorganisms12112143

**Published:** 2024-10-25

**Authors:** Huai Shi, Guohong Liu, Qianqian Chen

**Affiliations:** Institute of Resources, Environment and Soil Fertilizer, Fujian Academy of Agricultural Sciences, Fuzhou 350003, China; darkone@126.com (H.S.);

**Keywords:** *Shouchella clausii*, genome analysis, adaptation, antibiotic resistance, toxin, probiotic

## Abstract

*Shouchella clausii* (*S. clausii*) has been marketed as an important commercial probiotic, displaying significant therapeutic effects on antibiotic-associated diarrhea and providing benefits to humans. This study aimed to explore the distribution, adaptation, and probiotic properties of *S. clausii*. Based on 16S rRNA gene analysis, 43 strains of *S. clausii* were isolated from 317 soil samples in China. Based on the genomic index of Average Nucleotide Identity (ANI) results, 41 strains were confirmed as *S. clausii*, while two strains, FJAT-45399 and FJAT-45335, were identified as potential novel species distinct from *S. clausii*. Combined phenotypic and genomic predictions indicated that *S. clausii* could survive under harsh conditions. Comparative genomics revealed that these isolates possess antibiotic resistance genes, as well as capabilities for bacteriocin and folate production, while lacking toxins and hemolytic activity. Hemolysis tests indicated that strain FJAT-41761 exhibited non-pathogenic γ-hemolytic activity, while also demonstrating resistance to multiple antibiotics, consistent with probiotic characteristics. These findings suggest that strain FJAT-41761 is safe and holds potential as a future probiotic.

## 1. Introduction

*Shouchella clausii* (*S. clausii*) is a rod-shaped, spore-forming, Gram-positive, facultative alkaliphilic probiotic bacterium that can grow in both aerobic and anaerobic environments and colonize the gut. Based on genomic analysis, *S. clausii* belongs to the genus Bacillus rRNA group 6. Formerly known as *Bacillus clausii*, it was reclassified as *Alkalihalobacillus* by Patel and Gupta in 2019 [1], and subsequently reclassified as *Shouchella clausii* by Joshi et al. in 2021 [2].

*S. clausii* exhibits a range of functions, including the production of various enzymes such as alkaline protease, α-amylase, and endo-β-1,3-glucanase [3,4,5], as well as antioxidants [6] and biosurfactants [7]. Furthermore, it is capable of producing antibiotics [8] and degrading certain antibiotics, including cefuroxime, cefotaxime, and cefpirome [9]. These diverse functions make *S. clausii* suitable for a variety of applications. For instance, the solid-state fermentation of spent coffee grounds by *S. clausii* increases bioactive phenolic compounds and antimicrobial activities [10]. The co-culture of probiotic *S. clausii* T and *Bacillus amyloliquefaciens* HM618 can produce surfactin and effectively degrade antibiotics [11]. As an endophytic bacterium, *S. clausii* PA21 can utilize toxic glycoalkaloids (Gas) produced during improper storage of potatoes as the sole carbon source, thereby effectively degrading Gas and improving the storage characteristics of potatoes [12]. Additionally, *S. clausii*, in conjunction with *Bacillus thuringiensis*, has been used to reduce the alkali–silica reaction (ASR), thus preventing potential damage to concrete structures caused by ASR-induced expansion [13].

Probiotics are live microorganisms that, when administered in adequate amounts, confer health benefits to the host. One significant probiotic is *S. clausii*, which is marketed under multiple brands names, such as Enterogermina^®^ by Sanofi, and has been available for over 55 years [14]. *S. clausii* is unique due to its rapid growth in both aerobic and anaerobic environments. This probiotic supplement is increasingly used in both adult and pediatric populations. Previous studies have shown that *S. clausii* can effectively treat diarrhea, recurrent respiratory infections, and acute gastroenteritis [15,16,17].

Despite the significant interest in *S. clausii*, there have been few reports detailing its natural spread and adaptation, which are essential for its effective application as a probiotic and in various industrial processes. In this study, the authors investigate the distribution of *S. clausii* in China, analyze its adaptation, and evaluate its safety based on genome analysis and experimental findings.

## 2. Materials and Methods

### 2.1. Soil Samples and Isolates

A total of 317 soil samples were collected from 28 provinces and autonomous regions across China. These samples were obtained from a variety of habitats, including farmland, grassland, mountains, desert, Gobi, saline–alkali land, marine sediment, and microbial fermentation beds. Thin soil layers (0–20 cm) were packed in sterile bags, brought back to the lab, and stored at 4 °C. A 10 g portion of the soil was suspended in 90 mL of sterile water and shaken for 30 min. The blended soil slurry was boiled for 15 min at 80 °C to kill non-spore-producing bacteria and then diluted. The diluted marine sediments were spread on a 2216E plate and incubated at 30 °C for 2 days. Another set of diluted soil sample suspension was spread on an LB (Lysogeny Broth) plate and also incubated at 30 °C for 2 days. Single colonies were picked and purified until pure single colonies were obtained. These were then transferred into LB medium supplemented with 20% glycerol (*w*/*v*) and stored at −80 °C. All culture media were obtained from Shanghai Acmec Biochemical Technology Co., Ltd. (Shanghai, China).

### 2.2. Genomic DNA Extraction, 16S rRNA Gene Identification, and Genome Sequencing

Genomic DNA was extracted and purified from cultured cells using a DNA extraction kit (Shanghai Generay Biotech Co., Ltd., Shanghai, China), following the manufacturer’s instructions. The 16S rRNA gene was amplified and sequenced using primers and the conditions described previously [18]. The obtained 16S rRNA gene sequence was compared with available sequences of cultured species in the EZBioCloud server [19].

The draft genomes were sequenced at Beijing Novogene Bioinformatics Technology Co., Ltd. (China). Reads from each data set were filtered and high-quality paired-end reads were assembled using SOAPdenovo (version 2.04) [20]. The rRNAs and tRNAs were predicted using RNAmmer [21] and tRNAscan-SE, respectively [22]. Protein-coding regions were identified using Prodigal v.2.6.3 [23] with the “-p single” option and annotated against the Kyoto Encyclopedia of Genes and Genomes (KEGG) database [24]. All annotated genes were searched against the CAZy database [25]. Genes belonging to different carbohydrate-active enzymes or protease families were classified by in-house Python scripts according to the predictions. Each protein predicted by Prokka was annotated using BLASTP and Hmmscan against the Clusters of Orthologous Groups (COG) database and PFAM database with e-values < 1 × 10^−5^, respectively. Transporters were predicted by performing BLASTP with e-values < 1 × 10^−10^ using *S. clausii* protein sequences against all Transporter Classification Database (TCDB) sequences [26]. The ANI value was calculated by the OrthoANIu algorithm [27].

### 2.3. Adaptation Assessment of pH, Temperature and Salinity

To determine the range of adaptation to temperature, pH, and salinity, 43 strains of *S. clausii* were cultured under the following conditions. Pure colonies were inoculated on LB plates with pH 9 and then incubated at 10, 15, 20, 25, 30, 35, 40, 45, 50, 55, 60, and 65 °C for 2 days. After inoculating pure colonies on LB plates, the growth of the strain was observed in the range of pH 6–13 (interval of 1 unit) after incubation at 45 °C for 2 days. To determine the tolerance to salt, 11 different concentrations of NaCl were tested, including 0%, 1%, 2%, 3%, 4%, 5%, 6%, 7%, 10%, 15%, and 20% (*w*/*v*). Colonies were incubated on LB plates with pH 9 at 45 °C. The colony diameters were measured after 48 h to determine adaptation. The pH of the medium was adjusted using the buffer system as described by Narsing Rao et al. [28].

### 2.4. Phylogenomic Tree Construction

A total of 21 genomes of *S. clausii* were downloaded from the NCBI GenBank database and used to construct a phylogenomic tree along with the isolates from the present study. The core gene single-nucleotide polymorphism (cgSNP) was calculated using SNP-sites v2.5.1 [29]. A maximum likelihood (ML) phylogenetic tree was constructed based on cgSNP data using IQ-TREE v1.6.12 with 1000 bootstrap values [30]. Mash distance was also adopted to build a phylogenetic tree with UPGMA calculation using Usearch v11.0, with the parameters set as cluster_aggd -linkage avg -id 0.999 [31]. iTOL was used to visualize the phylogenetic trees online [32].

### 2.5. Degradation Ability Assays of Starch, Protein, and Cellulose

Screening of enzyme activities in *S. clausii* strains was performed according to González Pereyra et al. [33]. Alkaliphilic amylase, protease, and cellulase activities were tested on LB plates supplemented with starch (1%, *w*/*v*), casein (1%, *w*/*v*), and CMC-Na (1%, *w*/*v*), respectively. The pH of the above media was adjusted to pH 9.0. After spot inoculation, all plates were incubated at 30 °C for 2 days. The transparent zones around the colonies were observed after staining to determine the enzyme-producing capabilities. Starch, casein, and CMC-Na were all sourced from Shanghai Macklin Biochemical Technology Co., Ltd. (Shanghai, China).

### 2.6. Hemolysis Test

*S. clausii* strains were tested for hemolysis on LB agar supplemented with 5% (*v*/*v*) sheep blood by spotting fresh culture on the blood agar plates, followed by incubation at 30 °C under aerobic conditions for one week. The 5% sheep blood was commercially ordered from Solarbio Life Sciences (Beijing, China). Isolates that formed a clear zone and green halo around bacterial strains were assessed as β-hemolytic and α-hemolytic, respectively. Isolates without any clear transparent or greenish zone surrounding the colonies were denoted γ-hemolytic. Thus, the γ-hemolytic bacteria were considered non-hemolytic and then selected for antibiotic susceptibility testing [34].

### 2.7. Antibiotic Susceptibility Test

The disk agar diffusion method (Kirby–Bauer method) was employed to assess the sensitivity of potential probiotics to various antimicrobial agents. A sterile cotton swab was utilized to evenly distribute the *S. clausii* culture across the surface of an LB agar plate. Subsequently, sterile tweezers were used to place a drug-sensitive paper disk onto the agar surface. The plates were incubated at 30 °C for 1 day, after which the diameter of the inhibition zone was measured. The experiment was conducted in triplicate, and the average value was calculated to determine sensitivity according to the instructions provided with the drug-sensitive paper disks. A total of 18 antibiotics were selected for susceptibility testing, including cefazolin, ampicillin, chloramphenicol, amikacin, kanamycin, neomycin, gentamicin, rifampicin, doxycycline, tetracycline, vancomycin, penicillin, clindamycin, oxacillin, polymyxin B, streptomycin, azithromycin, and erythromycin. The drug-sensitive paper disks were procured from Tech-S (Guangzhou) Technology Service Co., Ltd. (Guangzhou, China).

### 2.8. Data Availability

The genomes supporting the results have been deposited at DDBJ/ENA/GenBank under the accession numbers from JACSJK000000000 to JACSNI000000000.

## 3. Results

### 3.1. Isolation and Identification of S. clausii

#### 3.1.1. Sample Collection and Preliminary Identification

From 317 soil samples collected across China, 43 potential *S. clausii* strains were identified through preliminary 16S rRNA gene sequencing. The 16S rRNA similarities between the 43 strains and the closest type strain, *S. clausii* DSM 8716T, ranged from 99.57% to 100%. The 43 strains were distributed across five provinces in China: Fujian, Heilongjiang, Qinghai, Sichuan, and Xinjiang (details in Table 1). They were found in various habitats, including volcanic valleys (2.3%), marine sediments (14.0%), microbial fermentation beds (MFB) (37.2%), mountains (18.6%), saline–alkali soils (11.6%), deserts (2.3%), grasslands (11.6%), and farmland (2.3%). The soil pH ranged from 5.8 (forest soil in Sichuan) to 8.7 (saline–alkali soil in Qinghai). This distribution indicates that *S. clausii* can survive in both acidic and alkaline environments.

#### 3.1.2. Genome and Phylogenetic Analysis

**Figure 1:**
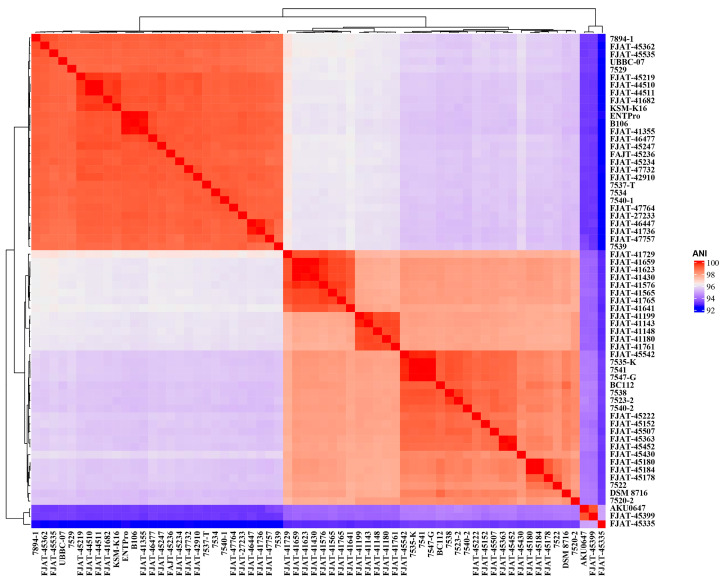
ANI indices of *S. clausii* isolates FJAT-45335, FJAT-45399, and AKU0647 compared to the type strain *S. clausii* DSM 8716T, suggesting two novel species. The colors indicate varying levels of the measured values, with red representing higher values and blue representing lower values.

The genomes of 43 isolated *S. clausii* strains were sequenced and assembled, and 21 genome sequences were obtained from the GenBank database (Table S1). The genome sizes of the 43 isolated strains ranged from 4.15 to 4.61 Mbp, and the DNA G+C content ranged from 44.18% to 44.74%. Due to the high 16S rRNA similarities between the 43 strains and the type strain *S. clausii* DSM 8716T, ANI was calculated based on genome sequences to determine the correct taxonomic status of the 43 potential *S. clausii* strains. ANI indices of our isolates FJAT-45335, FJAT-45399, and AKU0647 from NCBI with the type strain *S. clausii* DSM 8716T were all lower than the species threshold value of 95%, specifically 93.2%, 94.2%, and 94.1%, respectively (Figure 1, Table S2). We also found that the ANI between FJAT-45335 and FJAT-45399 was 93.2%, the ANI between FJAT-45399 and AKU0647 was 99.4%, and the ANI between FJAT-45335 and AKU0647 was 94.8%. Therefore, it was suggested that the three strains should be considered as two novel species different from *S. clausii*, and their taxonomic status will be confirmed using polyphasic taxonomy in the future. Other strains in this study were identified as *S. clausii* at the species level based on genome analysis (Table S2).

**Figure 2:**
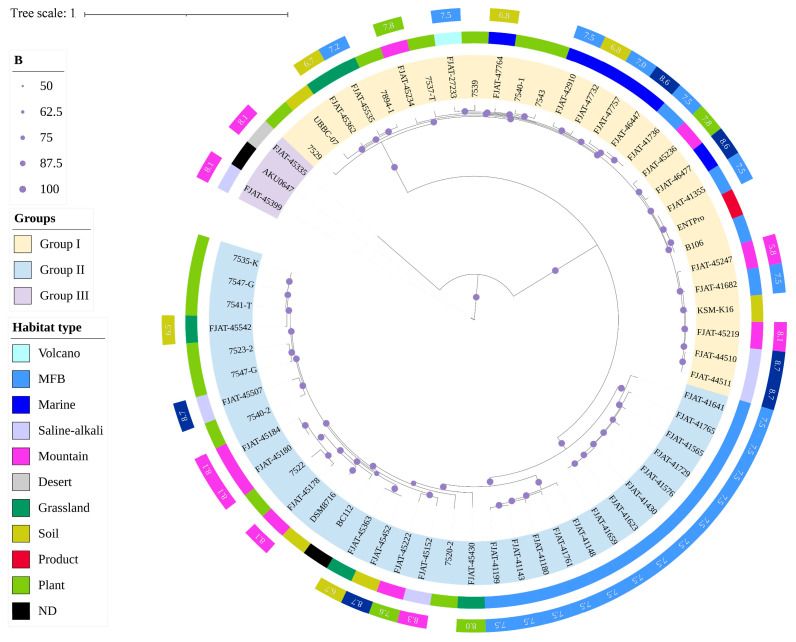
Maximum-likelihood phylogenomic tree of 64 *S. clausii* genomes based on core genome SNPs.

Comparative genomics was conducted to analyze the evolution and adaptation of *S. clausii* to diverse environments. A total of 64 genomes were analyzed to construct maximum-likelihood (ML) phylogenomic trees based on core genome SNPs (Figure 2). The habitat types of all strains were incorporated into the phylogenetic tree online using iTOL. It was suggested that *S. clausii* strains could survive in various environments, including terrestrial and marine ecosystems. The phylogenetic tree built using Mash distance supported the above result (Figure S1). Three groups were classified and well supported by bootstrap values. Based on ANI indices, Group III was studied as a potential novel species different from *S. clausii*. Therefore, Groups I and II were the main focus of subsequent studies. Group I comprises 18 isolates and 10 NCBI strains, with a sample pH range of 5.8 to 8.7. In contrast, Group II contains 23 isolates and 10 NCBI strains, with the majority of samples exhibiting pH values exceeding 7.5.

### 3.2. Environmental Adaptability of S. clausii

#### 3.2.1. Adaptability to Temperature, pH, and Salinity

To determine the growth properties of isolated *S. clausii* from different habitats, we performed large-scale phenotypic analysis and evaluated the growth ability of 43 isolates under varying levels of temperature, pH, and salinity. The results showed that *S. clausii* strains could grow across a wide range of temperatures, pH levels, and salinity (Table S3). In total, 88.4% of the strains grew well at a high temperature of 55 °C, and strain FJAT-45452 could grow at 60 °C. In total, 32.5% of the strains showed some biomass at 15 °C, and no strains could grow below 15 °C. Most strains grew between 20 °C and 55 °C, with optimum growth at 45 °C. For the pH test, it was revealed that all strains were alkaliphilic and survived at high pH levels, even at pH 13, whereas no growth was found at or below pH 6. The salinity test showed that most strains tolerated salinity concentrations of 0–10%. A total of 83.7% of the strains showed growth at 15% NaCl, and no strain could grow at 20% NaCl. The results suggest that *S. clausii* has strong adaptability to survive in various extreme environments, but no obvious relationship was found between growth conditions and source environments.

#### 3.2.2. Homeostasis in Extreme Environments by Genome Analysis

**Figure 3:**
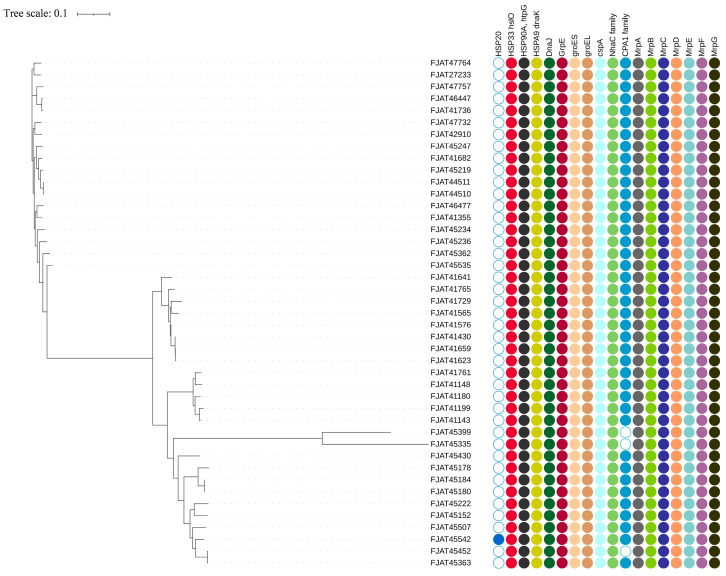
Heat shock and cold shock gene clusters and Na^+^:H^+^ antiporters identified in *S. clausii* genomes.

To explore how *S. clausii* survives in cold, hot, and alkaline environments, we selected cold shock and heat shock gene clusters, as well as Na^+^:H^+^ antiporters, as targets (Figure 3, Table S4).

There are three types of cold shock protein (Csp) genes that help adapt to cold environments [35], but only two copies of *cspA* were predicted in all isolates. This agrees with the above experimental result, as the strains could not grow below 15 °C. The *DesK*/*DesR* two-component system genes, which are responsible for maintaining cell membrane fluidity in low-temperature environments, were detected in all isolates.

The heat shock gene cluster *DnaK*-*DnaJ*-*GrpE* (KJE) chaperone system, along with the *GroEL*/*GroES* chaperone system, was identified in all isolates, facilitating their survival at elevated temperatures. Among the three related heat shock protein (Hsp) genes (HSP20, HSP33, and HSP90), only HSP20 was detected in the genome of strain FJAT-45542, whereas HSP33 and HSP90 were present in the genomes of all strains.

CPA1, CPA2, and CPA3 are the principal members of the large monovalent cation/proton antiporter (CPA) family [36]. CPA1 was identified in nearly all Group I and II isolates, while the more complex CPA3, which comprises a multicomponent structure with seven members, was found in two copies across all isolates. However, it lacked gene annotation related to the CPA2 family. Furthermore, the Na^+^:H^+^ antiporter family (*NhaC*) was detected in the genomes of all isolates, with two copies present in each genome.

#### 3.2.3. Carbon and Nitrogen Source Utilization Capacity

**Figure 4:**
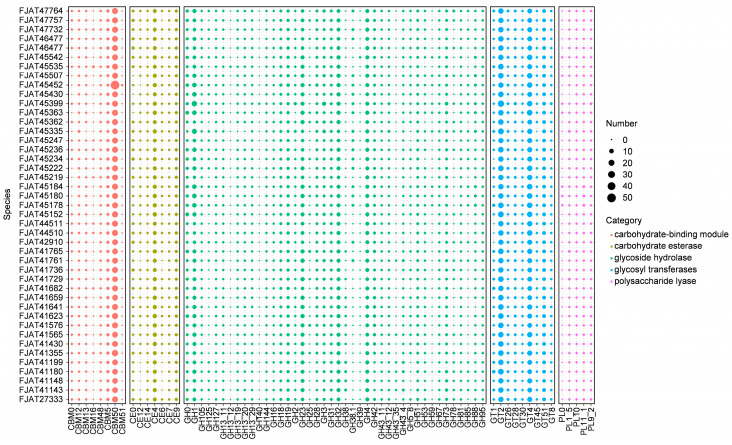
Distribution of carbohydrate-active enzymes (CAZymes) in *S. clausii* genomes. The number refers to the count of identified gene clusters.

To understand the genetic basis for the ability of *S. clausii* strains to survive in different niches, we first focused on genes involved in nutrient metabolism. Carbohydrate-active enzymes (CAZymes) involved in carbohydrate metabolism, including glycosyl transferases (GTs), glycoside hydrolases (GHs), carbohydrate esterases (CEs), carbohydrate-binding modules (CBMs), and polysaccharide lyases (PLs), were identified in all genomes of isolated strains. There were 6246 genes encoding the above enzymes: 28.5% for GT, 18.6% for CBM, 3.0% for PL, 10.4% for CE, and 39.6% for GH, indicating that GH accounted for the highest ratio (Figure 4, Table S5). GH18, GH19, CE4, and CBM50, which are involved in chitin degradation, showed significantly higher abundance in all isolates. The most numerous family involved in cellulose degradation was GH1 (β-glucosidases), with more than four genes per genome. Few genes were identified as belonging to the amylolytic enzyme representative family GH13, indicating that these strains possess little to no starch hydrolysis capability. The activity tests for amylase, protease, and cellulase indicated that all strains were capable of effectively hydrolyzing casein. Although none of the strains demonstrated the ability to degrade starch, some strains exhibited cellulose-degrading capabilities (Table S6), aligning with the predictions made from genomic analysis.

### 3.3. Probiotic Properties Through Genome Analysis

#### 3.3.1. Antibiotic Resistance in *S. clausii*

Genes encoding chloramphenicol O-acetyltransferase type A (*catA*) and chloramphenicol-sensitive protein (*RarD*) were found in several strains, indicating sensitivity to these antibiotics. Vancomycin resistance protein (*VanW*) and fluoroquinolone resistance proteins (*qnr* and *mcbG*) were present in all strains, indicating resistance to these antibiotics. The tetracycline resistance efflux pump *tet35* was present in all strains, but *tetM* and *tetO* were only predicted in strains FJAT-41143, FJAT-41148, FJAT-41180, FJAT-41199, and FJAT-41761, indicating resistance in these five strains. Penicillin-binding proteins were found in all strains, except gene *pbpC* was not found in FJAT-45452 and FJAT-45363.

Major Facilitator Superfamily (MFS) transporter genes *yitG*, *ymfD*, *yfmO*, and *marC*, which encode multiple antibiotic resistance proteins, were found in all strains. Additionally, multidrug resistance proteins of the DHA1 family (*bcr*, *blt*, *lmrP*, and *mdtG*) were identified in all strains. In contrast, the DHA2 family (*smvA*, *qacA*, and *lfrA*) was predicted only in FJAT-41430, FJAT-41565, FJAT-41576, FJAT-41623, FJAT-41641, FJAT-41659, FJAT-41729, FJAT-41765, FJAT-45152, FJAT-45222, FJAT-45452, FJAT-45507, and FJAT-45542, while DHA2 family members *lmrB* and *emrB* were found in all strains.

#### 3.3.2. Bacteriocins in *S. clausii*

The *nisA*-*G* gene cluster, involved in the synthesis and transport of lantibiotics, was identified in most isolates. Nearly all isolates contain the genes for the lantibiotic biosynthetic proteins *NisA*, *NisB*, and *NisC*. Additionally, sensor histidine kinases (*NisK*/*SpaK* and *NisR*/*SpaR*), which, together with the OmpR family, form a two-component system, are present in all isolates and play a crucial role in the activation and regulation of lantibiotic synthesis. Furthermore, the gene encoding the lantibiotic transport system (*NisE*-*F*) was detected in all isolates.

#### 3.3.3. Folate Biosynthesis Pathways in *S. clausii*

Folate production pathways in all strains were identified using the KEGG pathway database. Genome analysis revealed that almost all strains possessed the complete operon for synthesizing para-aminobenzoic acid (PABA). Moreover, all strains had the complete enzyme system for converting chorismate into PABA (*aroA*-*M*).

#### 3.3.4. Toxin Genes in *S. clausii*

To detect toxin genes in *S. clausii* strains, the bceT gene encoding single-component enterotoxin T and the *hbl* genes (*hblA*, *hblB*, and *hblC*) encoding hemolytic enterotoxin were predicted. Non-hemolytic enterotoxin genes, including *nheA*, *nheB*, and *nheC*, were also not observed in any of the isolates.

#### 3.3.5. Hemolysis Test Results

In this study, no obvious zone around the colonies was observed in any of the 43 strains of *S. clausii* after 3 days of incubation. However, most strains formed a green halo around their colonies and were considered α-hemolytic. A total of 8 strains had a clear zone around the colonies and were classified as β-hemolytic after 1 week of incubation. Among the β-hemolytic strains, FJAT-41576, FJAT-41180, and FJAT-41130 exhibited a very weak zone around their colonies. Only strain FJAT-41761, which did not show any clear transparent or greenish zone surrounding the colonies, was denoted γ-hemolytic at 3 and 7 days (Table S7).

## 4. Discussion

### 4.1. Strain Distribution and Genomic Analysis of Adaptability

*Shouchella clausii* exhibits a broad distribution in nature and demonstrates tolerance to various environmental conditions. The sources of isolation for the 73 *S. clausii* BioSamples registered on NCBI include soil, water, food, animal bodies, and feces from diverse environments. The diversity of microbial niches is attributed to their versatility and adaptability to fluctuations in temperature, nutrient availability, and pH levels. These heritable adaptations are influenced by the genome [37]. In this study, we analyzed the genomes of the isolated *S. clausii* strains, focusing on cold shock and heat shock gene clusters, as well as Na^+^:H^+^ antiporters, to elucidate their homeostasis mechanisms in extreme environments. Additionally, we examined their ability to metabolize complex carbohydrates.

Under cold conditions, low-temperature anteiso-branched fatty acids and Csps play a crucial role in stabilizing the fluidity of bacterial membranes [38]. The *DesK*/*DesR* two-component system is activated at low temperatures and regulates the synthesis of unsaturated fatty acids, thereby maintaining membrane lipid fluidity during temperature fluctuations [39]. Csps protect cells from the formation of mRNA secondary structures during cold shock, promoting efficient transcription and translation [40]. This cooperative mechanism enables *S. clausii* to effectively respond to low-temperature stress while maintaining its physiological functions and viability.

When cells are subjected to heat stress, HSPs are rapidly synthesized. Their primary function is to act as molecular chaperones, assisting other proteins in achieving proper folding and maintaining stability in high-temperature environments, thereby protecting cells from damage [41,42]. *DnaK* from the HSP70 family, *DnaJ* from the HSP40 family, and *GrpE* from the HSP22 family collectively form the KJE chaperone system, which plays a crucial role in bacterial protein homeostasis [43]. This system is regarded as a vital component of the smallest bacterial genomes and is ubiquitous across all bacteria (Warnecke, 2012). Additionally, *GroEL*/*GroES* can work in conjunction with *DnaK*/*DnaJ* to prevent protein misfolding in bacteria [44]. Members of the *Clp* protease family, particularly *ClpX*, *ClpB*, and *ClpG*, are essential in the bacterial response to high-temperature stress. They collaborate with HSP family members to enable bacteria to withstand heat stress through various mechanisms, including the degradation of unstable proteins and the promotion of protein remodeling [45]. In summary, the proteins encoded by these identified genes collectively form a complex molecular network that enables *S. clausii* to maintain protein homeostasis and function effectively in high-temperature environments through various strategies.

The Na^+^:H^+^ antiporter is critical for bacteria to sustain intracellular pH homeostasis and Na^+^ dynamic balance [46]. The presence of multiple Na^+^:H^+^ antiporter genes may enable *S. clausii* to maintain its osmotic and pH balance across various environments. Additionally, the two-component system *DesK* and *DesR* has been identified as a pH sensor. It was found to be incapable of activating *DesK* at low pH, irrespective of temperature [39]. In line with this, our findings indicate that all isolates were unable to grow at pH levels below 6.

We also focused on the genes associated with nutrient metabolism in *S. clausii* isolates, which provide the genetic foundation for their survival in various ecological niches. Within the animal gut, the capacity of microorganisms to metabolize complex carbohydrates is a crucial characteristic for their survival and competitive advantage; this capability also directly influences nutrient absorption and the overall health of the host [47]. To assess the potential of *S. clausii* isolates to degrade and metabolize complex carbohydrates and proteins, we conducted genomic analyses and evaluated the amylase, protease, and cellulase activities of the strains. The results indicate that while these isolates cannot utilize starch, they can effectively hydrolyze casein and cellulose, suggesting their potential to function as probiotics within the guts of animals.

### 4.2. Probiotic Properties

Probiotics function in decreasing gastrointestinal (GI) complications and modulating the immune system in the host. The basic important properties include tolerance to bile salts, antibiotic resistance, vitamin production, antimicrobial substance production, and toxigenic potential [48,49]. Based on our analysis of the genomes of *S. clausii* isolates, we propose that these strains possess promising probiotic properties.

Bile acid salts can induce intracellular acidification and disrupt biological membranes [50]. To survive in the human gastrointestinal tract, both commensal and pathogenic microorganisms must withstand the harmful effects of bile. Genome analysis revealed the presence of the Bile Acid: Sodium Symporter (BASS family, K03453), a family of proteins is responsible for the co-transport of bile acids and sodium ions across cell membranes, in all isolates examined in this study. Intriguingly, although not all organisms are capable of secreting bile, the BASS family is widely distributed across a variety of organisms, including microorganisms [51]. The presence of this protein may assist *S. clausii* in maintaining cell membrane integrity and transporting bile salts, thereby stabilizing the intracellular environment and enhancing survival in the intestinal milieu.

The drug-resistant properties of probiotics contribute to their survival during antibiotic treatment and the restoration of normal intestinal flora [52]. Studies have investigated the antibiotic resistance of *S. clausii* strains in commercial probiotic products through genome sequencing analysis, deeming them suitable for concurrent use with antibiotics [53,54]. However, there are relatively few analyses of potential candidate isolates. This study identified genes conferring resistance to antibiotics such as chloramphenicol, vancomycin, fluoroquinolones, tetracyclines, and β-lactams, as well as a significant number of MFS and other antibiotic efflux pumps, such as *tet35* (a tetracycline resistance efflux), present in the genomes of the majority of isolates. Furthermore, genome analysis revealed that the antibiotic resistance genes of *S. clausii* isolates were located on chromosomal DNA rather than on transposable elements. These results align with the conclusions of previous studies, indicating that *S. clausii* strains exhibit multiple antibiotic resistance properties; however, they do not have the capability to transmit these properties to other organisms [55,56].

Folate is an essential vitamin involved in human DNA replication, repair, and methylation processes, playing a significant role in human health, and it cannot be synthesized by human cells. One of the critical functions of probiotics is to provide additional supplements to the host through the production of folate [48]. Most reports on folate production originate from *Lactobacillus* and *Bifidobacterium*, with comparatively few studies focusing on Bacillus probiotics. Utilizing the KEGG pathway database, we found that nearly all isolates possess complete folate synthesis pathway genes, which is inconsistent with existing studies. It has been reported that certain commercial probiotic strains of *S. clausii* lack this complete system but include genes for the DHPPP biosynthetic pathway, including the gene encoding dihydropteroate synthase (EC 2.5.1.15), allowing them to produce folate in the presence of PABA supplementation [54].

It has been suggested that bacteriocins produced by Bacillus probiotics play a significant role in combating pathogens in the gut. Lantibiotics, which are cationic antimicrobial peptides synthesized by ribosomes, exhibit inhibitory effects on a variety of Gram-positive bacteria, including certain antibiotic-resistant strains [57,58]. *S. clausii* ENTPro, a commercial probiotic product, contains sactipeptides, gallidermin *BsaA2*, and lanthipeptide class I [54]. The majority of isolates in this study possess genes associated with the synthesis and transport of lantibiotics, suggesting their potential to inhibit enteric pathogens.

Although *S. clausii* is generally regarded as safe, there have been a few reported cases of bacteremia in immunosuppressed adults following its administration [59]. The potential of probiotics varies by strain; however, safety testing is a fundamental requirement for all probiotics. Only the strains that successfully undergo standardized safety and efficacy testing in accordance with regulatory guidelines are permitted for official use. Genome analysis, utilizing bioinformatics databases and practical software, has proven to be an effective approach for comprehensive safety pre-evaluations [60]. The results of toxin gene analysis indicated that none of the isolates contained the enterotoxin T, hemolytic enterotoxin, or non-hemolytic enterotoxin genes. This is consistent with previous research indicating that Bacillus strains do not produce B. cereus-like toxins [61]. Hemolysis indicates the presence of cytotoxic phospholipases associated with the virulence of a given bacterial strain [62]. Our results suggest that all isolates lack these genes, supporting their safety. Furthermore, hemolysis experiments revealed that, with the exception of eight strains, the majority of isolates exhibited low-pathogenic α-hemolysis, while strain FJAT-41761 displayed non-pathogenic γ-hemolysis, further corroborating the genomic analysis results.

In the antibiotics test, strain FJAT-41761 exhibited sensitivity to cefazolin, ampicillin, chloramphenicol, amikacin, kanamycin, neomycin, gentamicin, rifampicin, doxycycline, tetracycline, and vancomycin, while it was resistant to penicillin, clindamycin, oxacillin, polymyxin B, streptomycin, azithromycin, and erythromycin. Additionally, FJAT-41761 demonstrates growth at temperatures ranging from 20 °C to 55 °C, a pH range of 7 to 13, and tolerates 0% to 15% NaCl. This strain exhibits protease activity and possesses complete genes for the biosynthesis of folic acid and lantibiotics. These properties highlight the strain’s ability to survive in the gastrointestinal tract, antimicrobial activity, and beneficial interactions with host organisms, thereby indicating its potential as a probiotic.

## 5. Conclusions

This study represents the first comprehensive report on the distribution of probiotic *S. clausii* in various soil types, highlighting its higher prevalence in alkaline habitats. Out of the 43 potential isolates, 41 were confirmed as *S. clausii*, while 2 were identified as distinct species. Phylogenomic analysis suggests that the evolutionary trajectory of *S. clausii* may be more adapted to alkaline environments and reveals its potential mechanisms for tolerating various extreme conditions. The combined genome analysis and in vitro experiments indicate that strain FJAT-41761 exhibits promising probiotic properties, making it a potential candidate for future probiotic applications.

## Figures and Tables

**Table 1:** Isolation and geographic distribution of probiotic *S. clausii* strains.

Strain Code	Province	Site	Host	Habitat	Similarity (%)	Content (cfu/g)	Soil pH
FJAT-27233	Heilongjiang	Heihe Town	Grass	Wudalianchi Volcano	99.8	1 × 10^3^	7.5
FJAT-41143	Fujian	Fuqing Town	Microbial fermentation bed (MFB) of raising pig	MFB	99.9	13 × 10^5^	7.5
FJAT-41148				MFB	99.9	1 × 10^5^	7.5
FJAT-41180				MFB	99.9	4 × 10^5^	7.5
FJAT-41199				MFB	99.6	3 × 10^5^	7.5
FJAT-41355				MFB	99.6	1 × 10^5^	7.5
FJAT-41430				MFB	99.6	2 × 10^5^	7.5
FJAT-41565				MFB	99.6	4 × 10^5^	7.5
FJAT-41576				MFB	99.8	1 × 10^6^	7.5
FJAT-41623				MFB	99.6	1 × 10^6^	7.5
FJAT-41641				MFB	99.62	1 × 10^5^	7.5
FJAT-41659				MFB	99.6	1 × 10^5^	7.5
FJAT-41682				MFB	99.8	2 × 10^6^	7.5
FJAT-41729				MFB	99.6	2 × 10^6^	7.5
FJAT-41736				MFB	99.8	2 × 10^6^	7.5
FJAT-41761				MFB	99.9	1 × 10^6^	7.5
FJAT-41765				MFB	99.7	2 × 10^5^	7.5
FJAT-42910		Zhangzhou City	Soil	Marine sediment	99.9	1	7.5
FJAT-46447		Ningde City	Soil	Marine sediment	99.8	0.5 × 10^3^	8.64
FJAT-46477			Soil	Marine sediment	99.7	0.05 × 10^3^	8.64
FJAT-47732		Yunxiao County, Zhangzhou City	Mangrove	Marine sediment	99.7	0.5 × 10^3^	6.87
FJAT-47757			Mangrove	Marine sediment	99.8	0.5 × 10^3^	7.04
FJAT-47764			Mangrove	Marine sediment	99.9	0.5 × 10^3^	6.79
FJAT-44510	Qinghai	Qinghai Lake	Grass	Saline-alkali soil	99.9	20	8.73
FJAT-44511			Grass	Saline-alkali soil	99.8	10	8.73
FJAT-45152		Hoh Xil	Grass	Saline-alkali soil	100.0	10	8.26
FJAT-45178	Sichuang	MAO County, Aba Tibetan and Qiang Autonomous Prefecture	Soil	Mountains	99.6	0.01 × 10^3^	8.12
FJAT-45180			Soil	Mountains	99.7	0.1 × 10^3^	8.12
FJAT-45184			Soil	Mountains	99.6	0.1 × 10^3^	8.12
FJAT-45219			Soil	Mountains	99.8	60	7.62
FJAT-45222			Pine Tree	Mountains	100	10	7.84
FJAT-45234			Soil	Mountains	99.8	20	7.75
FJAT-45236			Soil	Mountains	99.6	20	7.75
FJAT-45247			*Loranthus* sp.	Mountains	99.8	0.1 × 10^3^	5.83
FJAT-45335	Xinjiang	Karamay	Gobi	Desert	99.9	0.01 × 10^3^	8.12
FJAT-45362		Xinyuan County, Yili Prefecture	Grass	Grasslands	99.8	0.04 × 10^3^	6.71
FJAT-45363			Grass	Grasslands	99.8	0.03 × 10^3^	6.71
FJAT-45535			Grass	Grasslands	99.9	0.56 × 10^3^	7.15
FJAT-45542			Grass	Grasslands	100.0	0.16 × 10^3^	6.45
FJAT-45430			Grass	Grasslands	99.8	0.01 × 10^3^	8.04
FJAT-45399		Zhungeer Basin	Soil	Saline-alkali soil	99.9	30	8.12
FJAT-45452		Fukang city, Changji Prefecture	Soil	Farm land	100.0	0.04 × 10^3^	8.71
FJAT-45507		Mulei County, Changji Prefecture	Grass	Saline-alkali soil	99.8	0.04 × 10^3^	8.66

## Data Availability

The original contributions presented in this study are included in the article/Supplementary Materials; further inquiries can be directed to the corresponding author.

## Supplementary Materials

The following supporting information can be downloaded at: https://www.mdpi.com/article/10.3390/microorganisms12112143/s1, Figure S1: Phylogenetic tree built using Mash distance; Table S1: The genome information of 43 isolated strains and 21 genomes of *S. clausii* downloaded from NCBI GenBank database; Table S2: ANI value between 64 strains; Table S3: The colony diameter (mm) of 43 isolated strains under different temperatures, pH, and salinity; Tabel S4: Genetic basis for *S. clausii* strains adaptation to diverse habitats; Table S5: CAZyme genes of 43 isolated strains; Table S6: The degrading ability of amylase, protease, and cellulase of 43 isolated strains; Tabel S7: Hemolysis test of 43 isolated strains using dot inoculation.
