# Supplementary figures and images for: Comparative Genomics and In Vitro Experiments Provide Insight into the Adaptation and Probiotic Properties of Shouchella clausii
Source: Microorganisms. 2024 Oct 25;12(11):2143. doi: 10.3390/microorganisms12112143 (PMC11596458; doi:10.3390/microorganisms12112143)

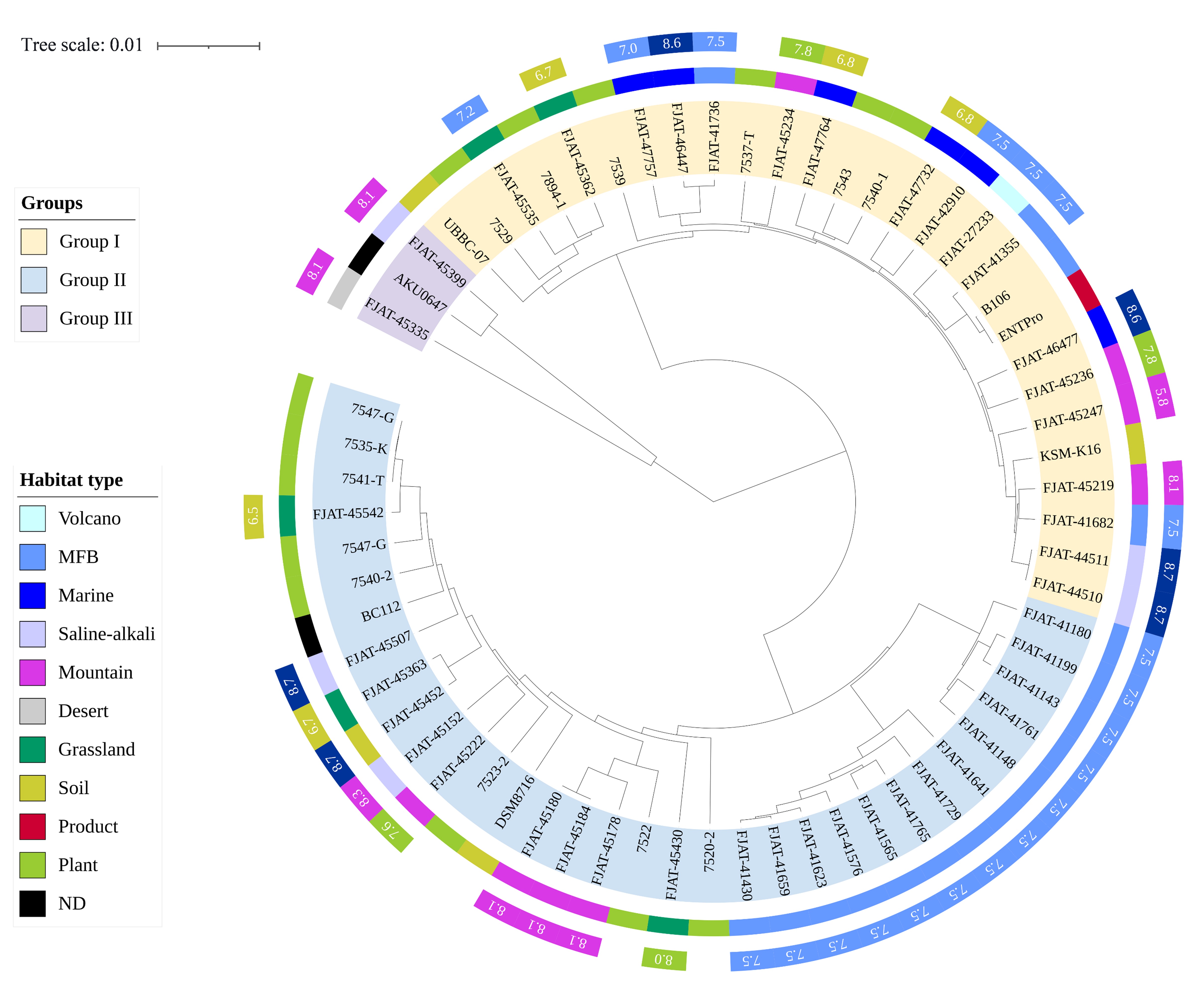

Supplement: Supplementary file 1 [file microorganisms-12-02143-s001.zip › Fig S1 Phylogenetic tree built using Mash distance.jpg]
